# Accuracy, User Acceptability, and Safety Evaluation for the FreeStyle Libre Flash Glucose Monitoring System When Used by Pregnant Women with Diabetes

**DOI:** 10.1089/dia.2017.0386

**Published:** 2018-03-01

**Authors:** Eleanor M. Scott, Rudy W. Bilous, Alexandra Kautzky-Willer

**Affiliations:** ^1^ Leeds Institute of Cardiovascular and Metabolic Medicine, University of Leeds, Leeds, United Kingdom.; ^2^ School of Medicine, Newcastle University Medicine Malaysia, Johor, Malaysia.; ^3^ Gender Medicine Unit, Division of Endocrinology and Metabolism, Department of Internal Medicine III, Medical University of Vienna, Vienna, Austria; and Gender Medicine Institute, Gars am Kamp, Austria.

**Keywords:** Diabetes, Flash glucose monitoring, Sensor, Pregnancy, Accuracy.

## Abstract

***Background:*** Accuracy of the FreeStyle Libre™ Flash Glucose Monitoring System has not been evaluated in pregnant women with diabetes. The aim of this study was to determine accuracy (compared to self-monitoring of blood glucose [SMBG]), clinical safety, and acceptability of the FreeStyle Libre System when used at home by this population.

***Materials and Methods:*** Seventy-four participants, with type 1 (T1D, *n* = 24), type 2 (T2D, *n* = 11), or gestational (*n* = 39) diabetes, were enrolled across 13 sites (9 in United Kingdom, 4 in Austria). Average gestation was 26.6 ± 6.8 weeks (mean ± standard deviation), age was 30.5 ± 5.1 years, diabetes duration was 13.1 ± 7.3 years for T1D and 3.2 ± 2.5 years for T2D, and 49/74 (66.2%) used insulin to manage their diabetes. Sensors were worn for up to 14 days. Sensor glucose values (masked) were compared with capillary SMBG values (made at least 4 times/day).

***Results:*** Clinical accuracy of sensor results versus SMBG results was demonstrated, with 88.1% and 99.8% of results within Zone A and Zones A and B of the Consensus Error Grid, respectively. Overall mean absolute relative difference was 11.8%. Sensor accuracy was unaffected by the type of diabetes, the stage of pregnancy, whether insulin was used, age or body mass index. User questionnaires indicated high levels of satisfaction with sensor wear, system use, and comparison to SMBG. There were no unanticipated device-related adverse events.

***Conclusions:*** Good agreement was demonstrated between the FreeStyle Libre System and SMBG. Accuracy of the system was unaffected by patient characteristics, indicating that the system is safe and accurate to use by pregnant women with diabetes.

## Introduction

The increased risks to both mother and developing fetus imposed on pregnancies complicated by diabetes are well established.^1–3^ The risks increase proportionately with worsening glycemic control.^4,5^ Effective glucose monitoring and therapy adjustments are recommended to protect against diabetes related pregnancy complications.^6^ Consequently, the glucose targets during pregnancy are more stringent than at other times and may predispose women to hypoglycemia. However, the condition of pregnancy also profoundly affects glycemic control and the management of diabetes—with increased glucose variability early in the pregnancy; progressively increasing insulin resistance, requiring intensification of therapy to maintain glucose targets; and increased risk of hypoglycemia (particularly during the first half of the pregnancy^7,8^), with more rapid onset of insulin-induced hypoglycemia.^2,9^ The risk of severe hypoglycemia is a major barrier to maintaining strict glycemic control, occurring up to five times more frequently during early pregnancy than in the period before pregnancy in women with type 1 diabetes (T1D).^10,11^ Women with gestational diabetes mellitus (GDM) have a particular challenge, since they are required to understand the disease, methods to monitor and control blood glucose, and the effects of diet, exercise, and medication on their glucose; all within a very short time frame to optimize their glucose control quickly through the rest of their pregnancy and minimize complications.

The National Institute for Health and Care Excellence (NICE) recommends that blood glucose levels are tested four to eight times daily to help achieve glucose targets; with more frequent testing for patients on an intensive insulin regimen and less frequent testing for patients on diet and exercise therapy, oral therapy, or single dose intermediate or long-acting insulin.^1^ Self-monitoring of blood glucose (SMBG) is commonly used for blood glucose monitoring during pregnancy and these test rates may be challenging to sustain throughout a pregnancy. In addition, the glucose variability and transient glucose excursions that may be experienced during pregnancy are not easily detectable using SMBG.

Continuous glucose monitoring (CGM) and other glucose sensor systems collect data at regular/short intervals, providing a fuller picture of the patient's glucose profile, rather than the discrete snap shots obtained from SMBG, without the pain and inconvenience of regular finger pricking. CGM is now widely recognized to provide improved clinical outcomes, such as reduced HbA1c, reduced time in hypoglycemia and hyperglycemia, and increased time in range, in nonpregnant adults with T1D and type 2 diabetes (T2D).^12–14^ However, the evidence to support the use and/or benefits of continuous sensing technology during pregnancy is more limited, and the published studies have reported mixed results on clinical outcomes for both mother and baby.^10,15–18^ Reported barriers to CGM use include technical challenges, calibration, skin irritation, frequent alarms (especially during sleep), differences between sensor and SMBG measurements, and cost.^10,17,19^ Despite the positive outcomes in the recent Continuous Glucose Monitoring in Pregnant Women with Type 1 Diabetes (CONCEPTT) study, more than 80% of the women experienced frustrations with CGM, such as connectivity issues, alarms, and calibration errors, and 48% of participants experienced skin reactions following sensor use.^20^ With the limited evidence of improved clinical outcomes, combined with the lack of published accuracy data for continuous sensing during pregnancy and the high cost of such systems, use of continuous glucose sensing is generally underutilized in the pregnant population.

The FreeStyle Libre Flash Glucose Monitoring System (Abbott Diabetes Care) is designed to replace SMBG testing for self-management of diabetes (except during rapidly changing glucose, to confirm hypoglycemia, or if symptoms do not match sensor results); it provides comprehensive glucose data (by measuring glucose in interstitial fluid [ISF], similar to CGM systems) and can be worn for up to 14 days, without the need for user (finger prick) calibration. The reader displays the glucose results after scanning over the small sensor worn on the back of the arm; it has no automatic alarms, but can alert the user to hypoglycemia or hyperglycemia and projected excursions when detected following a scan. At the time of the study, the system was approved for use in adults (with a limitation on use during pregnancy) and children. The aim of this study was to evaluate accuracy, safety, and user acceptability of the FreeStyle Libre Flash Glucose Monitoring System when used by pregnant women with diabetes.

## Materials and Methods

This prospective single arm study was conducted across 13 diabetes centers in the United Kingdom and Austria. The study (NCT02665455) was conducted in compliance with the study protocol and International Conference on Harmonisation Guideline for Good Clinical Practice. Competent Authority approval (Medicines and Healthcare Products Regulatory Agency [MHRA] and Bundesamt für Sicherheit in Gesundheitswesen [BASG]) and Ethics Committee approvals (including the Health Research Authority within United Kingdom) were obtained, and each participant gave written informed consent before participation in the study.

### Study participants

Women aged ≥18 years at ≥12 weeks gestation with a singleton pregnancy, with T1D, T2D, or GDM, and testing blood glucose at least twice per day were eligible to participate in the study. Exclusion criteria included concomitant disease or condition that may compromise patient safety; currently receiving or planning to receive dialysis treatment during the study, or moderate to advanced nephropathy; diabetic ketoacidosis in the previous 6 months; known or suspected allergy to medical grade adhesives; and experience of preeclampsia, HELLP (hemolysis, elevated liver enzymes, and low platelet count) syndrome, or prescribed tocolytic drugs for treatment of preterm labor during the current pregnancy. Any potentially eligible patient from the general diabetes population at each study site was invited to participate.

### Study design

There were three scheduled in-clinic visits. Visit 1 included data collection on demographics, current glucose management methods, prepregnancy glucose management methods (for participants with T1D or T2D), prepregnancy weight and HbA1c, previous pregnancies, and lifestyle; provision of a blood sample for HbA1c, fructosamine, and hematocrit measurement; height, weight, and blood pressure measurement; and sensor insertion and sensor application questionnaire. No device training was provided, other than instructions on how to use the device in masked mode and the product labeling. Adverse events (AEs) were reviewed at every participant contact.

Participants wore the sensor (on the back of their upper arm) for up to 14 days. Throughout this period, participants were asked to perform at least four premeal capillary blood glucose (SMBG) tests daily using the blood glucose strip-port on the reader (and FreeStyle Optium test strips; Abbott Diabetes Care), each immediately followed by an ISF glucose sensor measurement (data masked to participants) to allow comparison of results between sensor and SMBG.

At clinic visit 2 (between days 5 and 8) data from the device were uploaded, frequency of SMBG tests was reviewed, and any AEs experienced or concomitant medication changes were recorded.

During the final clinic visit (between days 12 and 15), the reader was unmasked for participants to experience full functionality of the system (for a short time while in-clinic), sensors were removed, data were uploaded, a blood sample was provided for HbA1c, fructosamine, and hematocrit measurement, user questionnaires were completed by the participant, and any AEs or concomitant medication changes were recorded.

Three sensor lots were used through the study, rotated within each study site, one lot per participant.

Participants were asked to maintain their preexisting diabetes management plan throughout their involvement in the study.

### Statistical analysis

Sensor values were compared with all temporally matched (within ±5 min) capillary blood glucose values, including those made in addition to the four premeal tests. The primary accuracy end point was the percentage of sensor results within Zone A of the Consensus Error Grid (ConEG) compared to SMBG results. A minimum sample size of 64 participants was required to detect a ≥5% difference in the mean percentage of paired points within Zone A of the ConEG, with a significance level of 5% and a power of 80%.

Other accuracy end points included ConEG and Clarke Error Grid analysis to evaluate the clinical accuracy of the system. Mean absolute relative difference (MARD), median absolute relative difference (median ARD), mean absolute difference (MAD), and mean relative difference (MRD) were used to evaluate analytical accuracy of the system. MAD and MARD were used to evaluate accuracy at low (<5.6 mmol/L [100 mg/dL]) and mid-to-high (≥5.6 mmol/L) glucose ranges, respectively. The proportion of results within ±1.1 mmol/L (20 mg/dL) of the SMBG value for glucose levels <5.6 mmol/L and within ±20% of the SMBG value for glucose levels ≥5.6 mmol/L (% within 1.1/20) was also used to evaluate overall analytical accuracy of the system. Multiway analysis of variance (ANOVA) was used to assess whether a range of factors affected sensor accuracy.

End points also included glycemic variability, where time in range, number and duration of hypoglycemic and hyperglycemic events, mean glucose, and standard deviation [SD] glucose were each determined for the overall study population and by the type of diabetes and diabetes management method. Time in range was defined for two separate analyses, as glucose results in the ranges 3.9–10.0 mmol/L (70–180 mg/dL) and 3.9–7.8 mmol/L (70–140 mg/dL), the tighter range aligning with NICE glucose targets for pregnant women 1 h after meals.^1^ A hypoglycemic event was defined for two separate analyses as excursions of at least 15 min below the target range (<3.9 mmol/L [70 mg/dL] and <3.0 mmol/L [54 mg/dL]), and a hyperglycemic event was defined for two separate analyses as excursions of at least 15 min above the target range (>10.0 mmol/L [180 mg/dL] and >7.8 mmol/L [140 mg/dL]).

Safety outcomes were analyzed for all enrolled participants, whether a sensor was worn or not.

All analyses were performed using SAS software, version 9.2 or later (SAS Institute, Cary, NC). Missing data were not imputed in the statistical analysis.

## Results

Eighty-three participants were enrolled in the study. Nine participants withdrew from the study on or before visit 1 and before sensor application: five participants chose to withdraw from the study (too busy or changed their mind); a further four participants were withdrawn by the investigator for a number of reasons (e.g., no longer met the inclusion/exclusion criteria, such as diagnosis of preeclampsia); no withdrawals were associated with use of the device. Therefore, 74 evaluable participants were included in the accuracy and glycemic variability analyses; all 83 participants were included in the safety analysis. Of the 74 evaluable participants, 49 (66.2%) were on insulin therapy, 18 (24.3%) were on diet only, and 7 (9.5%) were on metformin only; 39 (52.7%) had GDM, 24 (32.4%) had T1D, and 11 (14.9%) had T2D; and 29 (39.2%) were in the second trimester and 45 (60.8%) were in the third trimester of pregnancy. Of the participants on insulin therapy, 15 had GDM, 24 had T1D, and 10 had T2D. At baseline, participants' average age was 30.5 ± 5.1 years (mean ± SD), HbA1c was 40.2 ± 9.0 mmol/mol (5.8% ± 0.8%), fructosamine 223 ± 41 μmol/L, and hematocrit was 35.9% ± 2.7%. Other characteristics of the evaluable study participants, including a breakdown by diabetes type, are included in Table 1 (nonevaluable participants withdrew from the study before collection of demographic data).

**Table 1.** Baseline Characteristics of Study Participants

*Characteristic*	*Diabetes type*	*Mean ± SD*	*Median*	*Range*	N
Age (years)	Gestational	31.0 ± 4.9	31.0	21–41	39
	Type 1	28.4 ± 5.2	29.0	19–37	24
	Type 2	33.4 ± 3.7	33.0	27–39	11
	Overall	30.5 ± 5.1	31.0	19–41	74
Body mass index (kg/m^2^)	Gestational	34.2 ± 6.8	33.2	20.8–48.2	39
	Type 1	27.8 ± 4.5	27.7	19.5–35.1	24
	Type 2	35.9 ± 8.4	33.8	23.7–50.4	11
	Overall	32.4 ± 7.1	31.7	19.5–50.4	74
HbA1c (mmol/mol)	Gestational	35.3 ± 4.8	35.0	27–49	38^a^
	Type 1	48.3 ± 8.7	48.0	28–68	24
	Type 2	39.3 ± 8.5	39.0	31–61	11
	Overall	40.2 ± 9.0	38.0	27–68	73^a^
HbA1c (%)	Gestational	5.4 ± 0.4	5.4	4.6–6.6	38^a^
	Type 1	6.6 ± 0.8	6.5	4.7–8.4	24
	Type 2	5.7 ± 0.8	5.7	5.0–7.7	11
	Overall	5.8 ± 0.8	5.6	4.6–8.4	73^a^
Fructosamine (μmol/L)	Gestational	203 ± 17	198	171–249	38^a^
	Type 1	260 ± 48	255	198–380	24
	Type 2	212 ± 28	210	175–260	11
	Overall	223 ± 41	210	171–380	73^a^
Hematocrit (%)	Gestational	35.2 ± 2.3	35.8	29.6–39.1	38^a^
	Type 1	36.7 ± 2.9	36.2	32.4–43.7	24
	Type 2	36.5 ± 3.1	37.2	32.5–40.6	11
	Overall	35.9 ± 2.7	35.9	29.6–43.7	73^a^
Gestation (weeks)	Gestational	28.9 ± 5.2	29.0	14–36	39
	Type 1	25.2 ± 6.9	26.0	13–35	24
	Type 2	21.6 ± 8.7	18.0	13–35	11
	Overall	26.6 ± 6.8	28.0	13–36	74
Duration of diabetes (years)	Gestational	0.1 ± 0.1	0.1	0.0–0.5	39
	Type 1	13.1 ± 7.3	13.0	0.3–24.8	24
	Type 2	3.2 ± 2.5	3.4	0.2–8.3	11
	Overall	4.8 ± 7.3	0.3	0–24.8	74
BG tests/day (self-reported)	Gestational	4.7 ± 1.1	4.0	4–7	39
	Type 1	7.9 ± 1.8	7.5	5–12	24
	Type 2	4.8 ± 1.1	4.0	4–7	11
	Overall	5.7 ± 2.0	5.0	4–12	74

^a^ One participant with gestational diabetes does not have baseline laboratory results, since they were unable to provide a blood sample during visit 1.

SD, standard deviation.

**FIG. 1.**
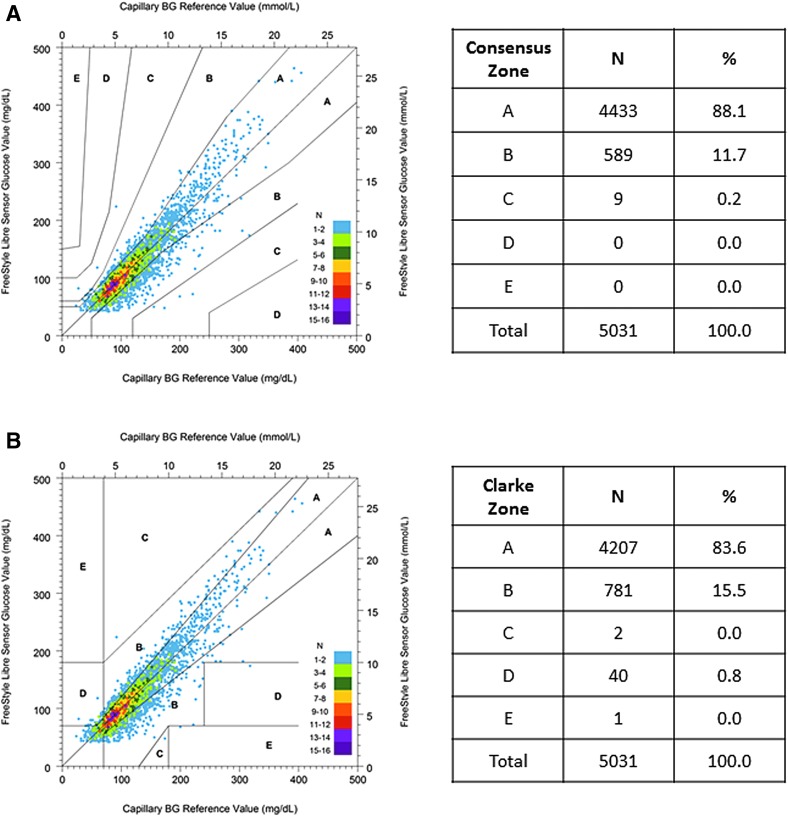
Consensus **(A)** and Clarke **(B)** error grid analysis comparing FreeStyle Libre sensor results to capillary BG results. BG, blood glucose.

There were 5031 paired capillary blood glucose to sensor glucose values used in the accuracy analyses. ConEG analysis demonstrated 88.1% of results in Zone A and 99.8% of results in Zones A and B (Fig. 1A) of the error grid. Clarke Error Grid analysis demonstrated 83.6% of results in Zone A and 99.1% of results in Zones A and B (Fig. 1B) of the error grid. Deming regression determined a slope of 1.12 and an intercept of −0.84 mmol/L (−15.1 mg/dL), with a correlation coefficient of 0.92. The overall MARD was 11.8%, median ARD was 9.5%, MRD was −1.1%, and the % within 1.1/20 was 87.1%. For paired results at lower glucose concentrations, with blood glucose <5.6 mmol/L (100 mg/dL) (*n* = 2133), MAD was 0.53 mmol/L (9.6 mg/dL); for those at higher glucose concentrations, with blood glucose ≥5.6 mmol/L (≥100 mg/dL) (*n* = 2898), MARD was 11.7%.

Accuracy of the system was the same with and without the rapidly changing glucose results included in the analysis (Table 2), indicating that the accuracy remains stable during times of rapidly changing glucose, such as in the postprandial state.

**Table 2.** Consensus Error Grid Analysis of Subgroup

	*Consensus zone*														
	*A*			*A and B*			*B*		*C*		*D*		*E*		
*Subgroup*	N	%	P	N	%	P	N	%	N	%	N	%	N	%	*Total* N
Rapidly changing															
All results	4433	88.1	N/A^a^	5022	99.8	N/A^a^	589	11.7	9	0.2	0	0.0	0	0.0	5031
Excluding results during rapidly changing glucose	4215	88.6		4752	99.8		537	11.3	8	0.2	0	0.0	0	0.0	4760
Diabetes type															
Gestational	2328	89.4	0.2985	2603	100.0	0.6128	275	10.6	1	0.0	0	0.0	0	0.0	2604
Type 1	1461	85.4		1704	99.6		243	14.2	6	0.4	0	0.0	0	0.0	1710
Type 2	644	89.8		715	99.7		71	9.9	2	0.3	0	0.0	0	0.0	717
Insulin use															
Insulin	2751	86.6	0.2351	3166	99.7	0.0702	415	13.1	9	0.3	0	0.0	0	0.0	3175
Noninsulin	1682	90.6		1856	100.0		174	9.4	0	0.0	0	0.0	0	0.0	1856
Trimester															
Second	1602	87.1	0.2723	1836	99.8	0.8504	234	12.7	4	0.2	0	0.0	0	0.0	1840
Third	2831	88.7		3186	99.8		355	11.1	5	0.2	0	0.0	0	0.0	3191
Age (years)															
<25	477	86.7	0.7625	549	99.8	0.7226	72	13.1	1	0.2	0	0.0	0	0.0	550
25–30	1503	86.8		1730	99.9		227	13.1	2	0.1	0	0.0	0	0.0	1732
31–35	1646	88.7		1852	99.8		206	11.1	3	0.2	0	0.0	0	0.0	1855
>35	807	90.3		891	99.7		84	9.4	3	0.3	0	0.0	0	0.0	894
Body mass index (kg/m^2^)															
<25	621	88.6	0.2770	699	99.7	0.2224	78	11.1	2	0.3	0	0.0	0	0.0	701
≥25 and <30	1268	88.5		1432	99.9		164	11.4	1	0.1	0	0.0	0	0.0	1433
≥30 and ≤35	1336	88.2		1512	99.8		176	11.6	3	0.2	0	0.0	0	0.0	1515
>35	1208	87.4		1379	99.8		171	12.4	3	0.2	0	0.0	0	0.0	1382
Sensor lot															
A	1439	88.6	0.1129	1622	99.8	0.1954	183	11.3	3	0.2	0	0.0	0	0.0	1625
B	1539	85.7		1794	99.9		255	14.2	1	0.1	0	0.0	0	0.0	1795
C	1455	90.3		1606	99.7		151	9.4	5	0.3	0	0.0	0	0.0	1611

^a^ Between-group comparison not performed since the set excluding results during rapidly changing glucose is a subset of all results.

The accuracy was similar across the subgroups, with a similar percentage of results in Zone A and in Zones A and B of the ConEG (Table 2)—there were no statistically significant differences (at a 5% level) in accuracy detected (ANOVA) for diabetes type, insulin versus noninsulin users, trimester, age group, body mass index (BMI), or sensor lot.

Glycemic variability of participants during their time in the study was determined from sensor data collected during the study. Duration within different glucose ranges and number of hypoglycemic and hyperglycemic events are broken down by diabetes type and insulin use (Table 3). As may be expected, time in range was lower for insulin using participants than noninsulin using participants and lower for participants with T1D than those with GDM and T2D; these were driven by the higher mean glucose for participants using insulin or with T1D.

**Table 3.** Time in Key Glycemic Ranges and Laboratory Parameters at Final Study Visit

			*Type of diabetes*			*Insulin use*	
n *(participants)*		*Overall 74*	*Gestational 39*	*Type 1 24*	*Type 2 11*	*Insulin 49*	*Noninsulin 25*
Sensor glucose							
Mean (SD)	mmol/L	6.17 (1.65)	5.39 (0.85)	7.63 (1.97)	5.76 (0.71)	6.60 (1.81)	5.32 (0.77)
	mg/dL	111.1 (29.7)	97.1 (15.3)	137.4 (35.5)	103.7 (12.7)	118.9 (32.6)	95.9 (13.9)
Time in range, hypoglycemia, and hyperglycemia, hours/day (24 h)							
3.9–10.0 mmol/L [70–180 mg/dL]	mean (SD)	18.9 (4.2)	20.6 (3.5)	15.6 (4.0)	19.9 (2.6)	17.6 (4.2)	21.4 (2.8)
3.9–7.8 mmol/L [70–140 mg/dL]	mean (SD)	16.4 (5.1)	19.1 (3.9)	11.6 (4.1)	17.4 (3.4)	14.4 (4.8)	20.3 (3.3)
<3.9 mmol/L [<70 mg/dL]	mean (SD)	3.26 (3.08)	3.12 (3.57)	3.43 (2.52)	3.36 (2.44)	3.69 (3.13)	2.42 (2.86)
<3.0 mmol/L [<54 mg/dL]	mean (SD)	1.06 (1.49)	0.79 (1.39)	1.51 (1.82)	1.02 (0.78)	1.38 (1.71)	0.44 (0.57)
>10.0 mmol/L [>180 mg/dL]	mean (SD)	1.88 (3.29)	0.30 (0.76)	4.98 (4.24)	0.73 (1.00)	2.75 (3.74)	0.18 (0.57)
>7.8 mmol/L [>140 mg/dL]	mean (SD)	4.33 (4.86)	1.75 (2.85)	9.00 (4.90)	3.29 (2.68)	5.90 (5.01)	1.26 (2.62)
Frequency of hypoglycemic and hyperglycemic events/day (24 h)							
<3.9 mmol/L [<70 mg/dL]	mean (SD)	1.86 (1.20)	1.75 (1.31)	2.01 (1.02)	1.94 (1.24)	2.06 (1.19)	1.48 (1.16)
<3.0 mmol/L [<54 mg/dL]	mean (SD)	0.69 (0.71)	0.54 (0.69)	0.91 (0.76)	0.73 (0.58)	0.87 (0.78)	0.33 (0.36)
>10.0 mmol/L [>180 mg/dL]	mean (SD)	0.80 (1.03)	0.23 (0.50)	1.79 (1.07)	0.64 (0.71)	1.13 (1.10)	0.14 (0.38)
>7.8 mmol/L [>140 mg/dL]	mean (SD)	1.81 (1.24)	1.22 (1.18)	2.62 (0.95)	2.11 (0.92)	2.26 (1.08)	0.93 (1.07)
Overall glucose variability							
Standard deviation	mmol/L	1.91 (1.09)	1.26 (0.33)	3.06 (1.17)	1.69 (0.37)	2.32 (1.13)	1.11 (0.20)
	mg/dL	34.4 (19.6)	22.7 (6.0)	55.2 (21.2)	30.5 (6.6)	41.7 (20.4)	20.1 (3.6)
Coefficient of variation, %		29.5 (9.2)	23.4 (4.8)	39.3 (7.4)	29.5 (5.3)	33.8 (8.2)	21.0 (2.7)
Laboratory parameters at final study visit^a^							
*n* (participants)		73^H^, 72^F^	38^H^, 37^F^	24^H,F^	11^H,F^	49^H,F^	24^H^, 23^F^
HbA1c	mmol/mol	40	36	48	39	43	34
	%	5.8	5.4	6.5	5.7	6.1	5.2
Fructosamine (μmol/L)		217	198	252	202	225	199
Hematocrit (%)		35.4	35.1	35.9	35.2	35.5	35.1

^a^ Only participants with laboratory parameters obtained at both baseline and visit 3 are included. One participant did not have baseline laboratory results (refer to Table 1). One participant did not have a fructosamine result for visit 3, since the laboratory was unable to analyze results: ^H^*n* for HbA1c and Hematocrit, ^F^*n* for Fructosamine.

**FIG. 2.**
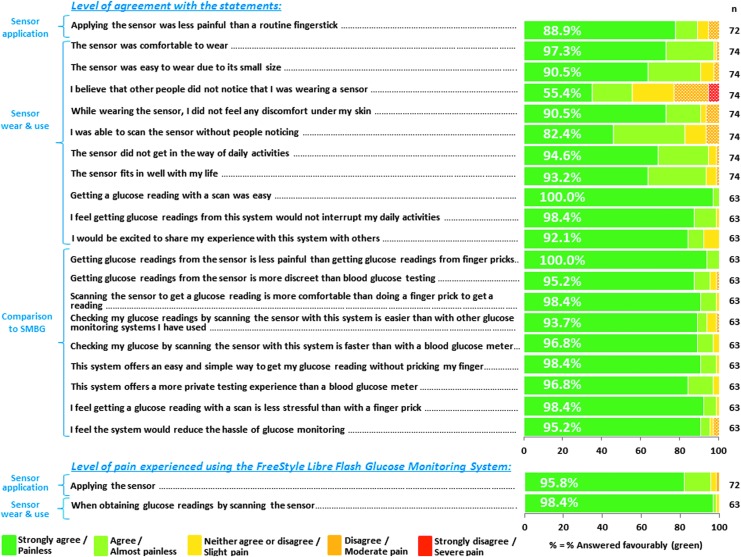
User acceptability questionnaire results.

Study participants completed a questionnaire (visits 1 and 3) and rated their experience with the system on a scale of 1 (strongly agree/painless) to 5 (strongly disagree/severe pain). Statements about sensor application (88.9%–95.8%), sensor wear and use (55.4%–100.0%), and comparisons to SMBG (93.7%–100.0%) were rated favorably (strongly agree/painless or agree/almost painless) by most participants (Fig. 2).

There were 27 AEs reported by 23 participants, none of which were related to the study device or study procedure. Of the 27 AEs, 4 were serious AEs (suspected preeclampsia, preeclampsia, vomiting, and decreased fetal movement). There were no unanticipated device related AEs. Five (6.8%) of the 74 participants reported experiencing 11 signs or symptoms associated with sensor application or insertion sites (bleeding, bruising, erythema, itching, and pain); all were mild in severity and resolved at study completion.

## Discussion

This study evaluated the performance and usability of the FreeStyle Libre System in pregnant women and is one of few glucose sensor accuracy studies for this population reported in the literature.^21,22^ The infrequent reporting of sensor accuracy studies involving pregnant women may be a result of few CGM systems being approved for use during pregnancy and some systems having a contraindication for use during pregnancy. At the time of the study, the FreeStyle Libre System had a similar contraindication. The study results demonstrated good agreement between the sensor glucose values and capillary blood glucose values, as illustrated through the different statistical and clinical accuracy measures reported, with comparable accuracy to that reported in the nonpregnant population.^23^ In a study of 72 adults with T1D and T2D using the FreeStyle Libre System, MARD was 11.4%, the percentage of results in Zone A and in Zones A and B combined of the ConEG was 86.7% and 99.7%, respectively, all compared to capillary blood glucose measurements. Use of capillary blood glucose as reference results allowed evaluation of real-life accuracy of the system under normal daily use, which would include fasting and postprandial periods around meals, impact of medication (including insulin), and sedentary and more active periods. This study has also confirmed that sensor accuracy is robust to patient characteristics such as age, BMI, insulin versus noninsulin use, diabetes type, and also to the stage of pregnancy. Similar observations about the system being robust to a number of patient variables have been made in other studies using the FreeStyle Libre System in nonpregnant participants.^23,24^ Accuracy of the FreeStyle Libre System, in combination with its robustness to differing patient characteristics, is sufficient to recommend its use to pregnant patients as support in optimizing their glycemic control and achieving the tight glucose control needed during pregnancy.

The system is unique among current glucose sensing technology options in that it does not require user calibration and it can be worn for up to 14 days. There are limited published data to allow comparison of the accuracy of the FreeStyle Libre System in this study to that for other continuous glucose sensors. However, the relationship between average glucose and HbA1c is similar to that reported for pregnant women with T1D and T2D using conventional CGM.^25^ One study reported accuracy of the FreeStyle Navigator CGM during exercise for pregnant subjects with T1D, with a median ARD of 11.8% and 96% of results within Zones A and B of the Clarke Error Grid at rest compared to venous blood glucose results (352 paired sensor-blood glucose results).^21^ Accuracy was lower during exercise. Another study in pregnant women with T1D also reported on the accuracy of the Freestyle Navigator CGM,^22^ with MARD and median ARD of 13.3% and 11.4% (*n* = 1923), respectively, compared to venous plasma glucose. Although the two studies were very different in design to the current study, the results provide an indication that the accuracy of the factory calibrated FreeStyle Libre System (99.1% of results within Zones A and B of Clarke Error Grid, MARD 11.8%, and median ARD 9.5% for overall population) compares well with that for CGM systems requiring user (finger prick) calibration. As noted in other publications, the lack of user calibration eliminates potential variations in sensor systems that may be introduced through errors in SMBG results used for calibration, calibration at inappropriate times (during rapidly changing glucose), missing calibrations, or use of sensor glucose rather than SMBG values for calibration.^23,24,26–28^

The masked sensor data collected throughout the study provided a snapshot of the time the participants from the United Kingdom and Austria spent in key glycemic ranges during their 2 weeks in the study, throughout which the participants were using SMBG to manage their diabetes. NICE recommends that patients on insulin are advised to maintain blood glucose levels >4 mmol/L (72 mg/dL).^1^ The insulin using participants in this study were not meeting this glucose target, with glucose levels <3.9 mmol/L (70 mg/dL) for 3.7 h per day. Other glucose targets recommended by NICE relate to fasting (<5.3 mmol/L [95 mg/dL]) and postmeal targets (<7.8 mmol/L [140 mg/dL] 1 h after meals or <6.4 mmol/L [115 mg/dL] 2 h after meals) and apply equally to patients with T1D, T2D, and GDM.^1^ While these aren't specifically evaluated in this study, the time participants spent with glucose >7.8 mmol/L (140 mg/dL) was 4.3 h per day on average for the full study population and as high as 9.0 h per day on average for the T1D subgroup and 5.9 h per day on average for the insulin using subgroup, suggesting that postmeal targets were also not being met. Although these data only represent a 2-week period, it suggests that there is significant opportunity to improve glycemic control in pregnant women with diabetes.

SMBG provides single, intermittent glucose values, which may not capture transient glucose excursions caused by the increased glucose variability experienced during pregnancy. In comparison, the FreeStyle Libre System displays the current glucose value, the last 8 h of glucose data, and trend information each time the sensor is scanned; providing significantly more data to guide treatment decisions for this challenging population than SMBG. While the system does not provide automated alarms for hypoglycemia and hyperglycemia, it does provide screen alerts when hypoglycemia and hyperglycemia are detected, or when glucose is projected to be hypoglycemic or hyperglycemic within 15 min, following a scan. Other studies have demonstrated that use of the FreeStyle Libre System can significantly reduce the amount of time spent in hypoglycemia.^13,14^ Further studies are needed to confirm whether the same improvement can be achieved with its use during pregnancy.

The study has demonstrated safety and user acceptability of the FreeStyle Libre System among a pregnant population. Similar to a study performed in nonpregnant adults, there were no unanticipated device related AEs.^23^ Anticipated signs or symptoms associated with sensor application or insertion sites (bleeding, bruising, erythema, itching, and pain) were reported for 7% of participants, a lower rate than observed in previous studies where nonpregnant adults (36%) and children and young people (44%) used the system.^23,24^ The user experience questions were rated favorably by most participants following sensor application, sensor wear and use, and in comparison to SMBG testing. Other studies have associated lack of improvement in glycemic control to reduced sensor wear and poor adherence to CGM.^29–33^ The high user acceptability demonstrated in this study (and in other studies following extended use of the FreeStyle Libre System^13,14^), together with the simplicity of the system and absence of alarms, may imply higher user compliance when using this system than has been observed in CGM studies in pregnancy.^10,15,18,19^

Limitations of this study are that the study design was not powered to compare accuracy between different subgroups and that a mixed cohort was used. Longer term studies, using the full functionality of the system (unmasked), are required to evaluate whether this system can provide improved adherence with sensor wear and improved glycemic and pregnancy outcomes for mother and baby. Such studies could evaluate women with different types of diabetes separately to determine whether improvements in glycemic control and pregnancy outcomes can be achieved in T2D and GDM in addition to T1D.

## Conclusions

The accuracy, safety, and user acceptability of the FreeStyle Libre System for women with diabetes during pregnancy have been demonstrated. Accuracy was unaffected by the type of diabetes (T1D, T2D, GDM), whether or not insulin was used, the stage of pregnancy, the age or BMI of the participants, thus making the system suitable for women with diabetes during pregnancy. It is anticipated that the provision of comprehensive glucose data for up to 14 days, from a system that is easy to use, could support enhanced diabetes management during pregnancy. In addition, the reduced pain and burden for the user (since finger-stick calibration is not required) and lack of alarms may support more extensive use of glucose sensing, since these were two of the barriers to more extensive CGM use reported in previous studies evaluating CGM use during pregnancy. Further studies are needed to investigate whether improved glycemic control and pregnancy outcomes for the mother and baby can be achieved with prolonged use of the FreeStyle Libre System during pregnancy.
